# Leptin Levels in Various Manifestations of Pulmonary Tuberculosis

**DOI:** 10.1155/2007/64859

**Published:** 2007-01-04

**Authors:** Hakan Buyukoglan, Inci Gulmez, Fahrettin Kelestimur, Levent Kart, F. Sema Oymak, Ramazan Demir, Mustafa Ozesmi

**Affiliations:** ^1^Department of Pulmonology, Faculty of Medicine, Erciyes University, 38200 Kayseri, Turkey; ^2^Department of Endocrinology and Metobolism, Faculty of Medicine, Erciyes University, 38039 Kayseri, Turkey; ^3^Department of Pulmonology, Faculty of Medicine, Karaelmas University, Kozlu, 67600 Zonguldak, Turkey

## Abstract

*Background*. Proinflammatory cytokines are prime candidates as causative agents of the metabolic changes that eventually result in tuberculosis-associated weight loss. Microbial products and cytokines such as TNF and IL-1 increase leptin expression dose dependently in adipose tissue. Leptin plays an important role in cellular immunity. *Objectives*. In this study, we investigated serum leptin and TNF-*α* levels before and after antituberculosis therapy in patients with active pulmonary tuberculosis (TB). *Methods*. Twenty five in patients with active pulmonary TB and 18 healthy controls participated in the study. Leptin and TNF-*α* levels were measured before treatment and six months after the treatment and they were compared with the control group. Body mass index (BMI) and chest X-rays before and after the treatment were also evaluated. *Results*. The leptin levels before and after the treatment were 1.66±1.68 ng/mL and 3.26±3.81 ng/mL, respectively. The leptin levels of tuberculous patients were significant than in healthy patients (*P* < .05). The BMI was 19.36 ± 2.55 kg/m^2^ before the treatment and 22.87 ± 3.13 kg/m^2^ after the treatment. The TNF-*α* level was 23.19±12.78 pg/mL before the treatment and 15.95±6.58 pg/mL after the treatment. There was no correlation between leptin and TNF-*α* levels. Leptin levels were low in patients who had sequela lesion on chest radiographs. *Conclusion*. Leptin levels are suppressed in tuberculous patients and low leptin levels may contribute to increased susceptibility to infection and recovery with sequela lesions.

## 1. INTRODUCTION

Tuberculosis (TB) is a major cause of death around the world, with most of the 1.5 million deaths per year attributable to the disease occurring in developing countries [1]. One of the most important and common complaints of tuberculous patients which also affects the immune status is weight loss. Antimycobacterial treatment often increases weight but patients may remain underweight even six months after the successful chemotherapy. Although wasting is probably one of the determinants of the disease severity and outcome, the pathogenesis of tuberculosis-associated wasting is largely unknown [2].

Some cytokines promote inflammation and are called proinflammatory cytokines (tumor necrosis factor-alfa (TNF-*α*), interleukin (IL)-1, IL-6, and IL-8), whereas other cytokines suppress the activity of proinflammatory cytokines and are called anti-inflammatory cytokines (IL-4, IL-10, and IL-13) [3]. Proinflammatory cytokines are prime candidates as causative agents of the metabolic changes that eventually result in tuberculosis-associated weight loss. TNF-*α* has some harmful effects, such as acute-phase pathophysiologic events including fever and tissue necrosis. It also plays a protective role against mycobacterial infection [3, 4].

Leptin, the product of the “ob” gene, regulates food intake and energy expenditure. In humans, circulating leptin levels are increased in obesity and are regulated by fasting, feeding, and body weight changes [5–7]. In addition to playing a role in energy regulation, leptin also regulates endocrine and immune functions [6–10]. Leptin links the proinflammatory T-helper 1 immune response to the nutritional status and the energy balance. It has been suggested that leptin mediates anorexia in chronic inflamatory states [11].

The relationship between leptin and pulmonary tuberculosis is not completely understood. There are very few studies relating the level of leptin and TNF-*α* before and after antituberculosis therapy and their results are contradictory. For this reason, we have decided to investigate the role of leptin and TNF-*α* in tuberculosis.

## 2. MATERIALS AND METHODS

Twenty five inpatients (19 males (mean ± SD age, 35.47 ± 13.91 years) and 6 females (mean ± SD age, 37.66 ± 18.20 years)) with active pulmonary TB (mean ± SD age, 36.0 ± 14.66 years) and 18 healthy controls 10 males (mean ± SD age, 38.60 ± 15.57 years and eight females (mean ± SD age 28.75 ± 5.59 years)) participated in the study. All patients were recruited from the Erciyes University Medical School, Outpatient Clinic of Pulmonology. On entry, all patients had positive smear for acid-fast bacilli in sputum or bronchial lavage and subsequent cultures of these specimens yielded tubercle bacilli. None of the patients had any evidence of concomitant bacterial or viral infections as indicated by sputum and blood cultures and viral serologic study including HIV. All patients were administered anti-TB therapy in which isoniazid (H), rifampicin (R), pyrazinamide (Z), and streptomycin (S) or ethambutol (E) were used. All the patients with TB were treated with standard therapy for active TB regimen which consists of 2 months of initial phase of HRZ E/S followed by a 4-month continuation phase of HR. Dosage recommendations of treatment of TB were H 5 mg/kg, maximum 300 mg, R 10 mg/kg maximum 600 mg, Z 15–30 mg/kg maximum 2 g, E 15–25 mg/kg maximum 1500 mg after 1 month reduced to 1000 mg, S 15 mg/kg maximum 1 g.

All patients had pulmonary symptoms such as cough, fever, and hemoptysis compatible with TB. To assess the presence, form, spread, and size of the TB cavities and infiltrations in the lungs, all 25 patients received plain posteroanterior and lateral chest X-rays. To avoid observer bias, three pulmonary physicians initially assessed the X-rays independently. The patients were divided radiologically into three categories; minimal, moderate-advanced, and far-advanced. In minimal TB, the lesions include those of slight to moderate density but they do not contain demonstrable cavitations. They may involve a small part of one or both lungs, but the total extent, regardless of distribution, should not exceed the volume of the lung on one side that occupies the space above the second condrosternal junction and the spine of the fourth thoracic vertebra or the body of the fifth thoracic vertebra. Moderate-advanced TB lesions include disseminated lesions of slight to moderate density that may extend throughout the total volume of one lung or the equivalent in both lungs; dense or confluent lesions are limited in extent to one third of the volume of one lung; and total diameter of cavitations, if present, must be less than 4 cm. In far-advanced TB, total cavity diameters are more than 4 cm and lesions are more extensive than moderately advanced [12].

Additionally, patients were assessed for loss of appetite, weight loss, and any other weaknesses. Body mass index, erythrocyte sedimentation rate (ESR), and body temperature were measured initially and after therapy. Body mass index (BMI) was measured as body weight (kg)/square of height (m^2^). A control group of 18 healthy volunteer subjects (mean age was 34.99 ± 12.7 years) was also studied. We also evaluated normal volunteers with chest X-rays, routine laboratory tests, and physical examinations. None of the control subjects had any evidence of infection and systemic disorders. All patients and volunteers gave informed consent and the Erciyes University Medical School, Ethics Committee, approved the protocol.

### 2.1. Immunoassays

All serum samples were collected from the patients and the control group before and after the 6 months of anti-TB therapy. Serum samples for the measurement of leptin and TNF-*α* level were drawn between 8^00^−10^00^ hours and were preserved at −70°C. All samples were assigned code numbers and were processed by an investigator. Serum leptin was determined using a commercially available radioimmunoassay kit (DsLAB Inc., Tex, USA) and TNF-*α* using enzyme-linked immunosorbent assay kits (Camarillo Inc., Calif, USA). The intra-assay level of leptin was 2.75±0.10 ng/mL ((coefficients of variation (CV) 3.7%)) and interassay level of leptin was 2.88 ± 0.19 ng/mL (CV 6.6%). The sensitivity of TNF was 6 pg/mL (interassay CV: < 7.8% and intra-assay CV: < 7.2%). All samples were assayed in duplicate. These parameters were measured in patients after 6 months of anti-TB treatment.

### 2.2. Statistical analysis

The Kolmogorov-Smirnov test was used for confirmation of the parameters. All figures are presented as mean ± SD. *T* tests and ANOVA were used for comparisons between the groups. Spearman's rank correlation was used to examine the relationship between the expression individual cytokines and clinical parameters. All *P* < .05 were considered significant. Stastics were calculated with SPSS software, version 10.0 (SPSS Inc., Chicago, Ill, USA; http://www.spss.com).

## 3. RESULTS

Demographic data of the patients are summarized in Table 1.

The mean ages of the patients and the controls were similar (36.0±14.66 versus 34.22±12.9, resp.). Before treatment, the BMI of the patients was significantly lower than the BMI of the controls (19.36±2.55 kg/m^2^ versus 24.23±3.56 kg/m^2^, resp.; *P* = .001). After anti-TB therapy, the BMI of the patients increased significantly and was similar to BMI of the control subjects.

The mean serum leptin levels of the patients before treatment were 1.66 ± 1.68 ng/mL, while the mean serum leptin levels of the controls were 15.74 ± 9.12 ng/mL, and the difference between the two groups was statistically significant (*P* = .001). Treatment resulted in an increase in serum leptin levels (3.26 ± 3.81 ng/mL) but postreatment leptin levels of the patients were still significantlly lower than leptin levels of the controls (*P* = .001). Although female patients had higher pretreatment leptin levels (2.17 ± 0.64 versus 1.50±1.88 ng/mL) and higher posttreatment leptin levels (4.38±2.95 versus 3.18±4.26 ng/mL) than the male patients, they were not statistically significant.

Serum TNF-*α* levels before treatment were higher in the patients than in the controls (23.19 ± 12.78 versus 14.22 ± 7.17 pg/mL, resp.; *P* < .05). Postreatment TNF-*α* levels were similar in both groups. There was no significant association between leptin and TNF-*α* concentrations. No correlation was found between BMI, body temperature, TNF-*α*, and leptin levels before treatment (*r* = 0.03, *r* = 0.33, *r* = −0.15). Leptin and TNF-levels are shown in Table 2.

The concentrations of TNF-*α* and leptin in the patients according to chest X-ray findings are presented in Table 3.

After treatment, there was a substantial increase in serum leptin concentrations in minimal and moderate-advanced cases. However, in all the three groups of patients, there were no statistically significant differences in serum TNF-*α* and leptin levels before and after the therapy.

The patients with sequelae lesions had lower leptin concentrations after anti-TB therapy (2.13 ± 1.96) than the patients with no squelae lesions (6.9 ± 5.72 ng/dL, *P* < .05) but the concentrations of TNF-*α* and BMI between the groups were similar (Table 4). There were no statistically significant differences in these parameters between the patients with cavity and without cavity. In the patients, there was no correlation for TNF-*α* and leptin levels with body mass index, age, fever, and ESR. There was no statistically significant difference between the extent of lesions and age.

## 4. DISCUSSION

Tuberculosis symptoms may be divided into two categories, systemic and pulmonary. Cough, anorexia, and weight loss are the most common symptoms of TB. The systemic symptom most frequently observed is a low-grade fever. Other systemic symptoms such as sweating (night sweat), weakness, fatigue, and headache may be present [13].

The anorexia during infection is part of the generalized host defense reaction. Despite being beneficial in the beginning, persistent anorexia delays recovery and is ultimately deleterious. Microbial products such as bacterial cell wall compounds (e.g., lipopolysaccharides), microbial nucleic acids (e.g., DNA, RNA), and viral glycoproteins cause the anorexia during infections. Microbial products stimulate the production of proinflamatory cytokines (e.g., ILs, TNF-*α*). Several cytokines reduce food intake after parenteral administration, suggesting that their enhanced production in respons to microbial products contributes to the anorexia of infection [14].

TNF-*α* induces fever and weight loss, which are typical symptoms of TB [15]. It has been shown to be associated with both protection and pathogenesis in mycobacterial infections [15, 16]. Several studies have shown that the systemic administration of recombinant TNF increases leptin gene expression from fat tissue [17, 18]. We found higher levels of TNF-*α* and low levels of leptin in TB patient group compared to control indicating no significant association between these two parameters.

Recent findings support the hypothesis that cytokine induction of leptin may play a significant role in anorexia and cachexia of inflammatory diseases and cancer. Microbial products and cytokines such as TNF and IL-1 increase leptin expression dose dependently in adipose tissue [18]. The cytokine-induced increase in leptin expression or its serum levels and reduction in food intake are closely related. Acute inflammatory stimuli such as surgical cholecystectomy have been shown to increase leptin levels in humans [19]. However, in chronic inflammatory and infectious diseases such as AIDS, rheumatoid arthritis, and inflammatory bowel disease, no changes in leptin levels have been observed [20–22]. Çakir et al. [23] have found increased levels of TNF-*α* and leptin in tuberculosis patients with good correlation of these two parameters and interpreted that the eleveted leptin level leads to weight loss, and may therefore contribute to the inflammatory process. Conversely, we found reduced leptin levels in TB patients both before and after the treatment. Although previous data have shown that plasma leptin levels are associated with loss of appetite in pulmonary tuberculosis, we were unable to demonstrate significant correlation between plasma leptin levels and loss of appetite [24].

Initially, leptin was considered as an antiobesity hormone, but experimental evidence has also shown pleiotropic effects of this molecule on hematopoiesis, angiogenesis, and T lymphocyte functions [5, 7, 9]. Leptin belongs structurally to the long-chain helical cytokine family such as inter-IL-2, IL-12, and the growth hormone [8]. Leptin links the proinflammatory Th1 immune response to the nutritional status and the energy balance [8, 11]. Ob/ob mice display immune defects with lymphoid organ atrophy, mainly affecting thymic size and cellularity [25]. Leptin deficiency is responsible for immunosuppression and reduced T cell response to microorganisms [9, 24] Leptin replacement reverses the immunosuppressive effect of acute food deprivation in mice [25].

In a recent study, morbidly obese children, who were congenitally deficient in leptin, have reduced number of circulating CD4 T cell and impaired T cell proliferation and cytokine release, which were reversed by daily subcutaneous injections of recombinant human leptin [26]. Leptin levels were found to be elevated in patients with sepsis and low leptin levels in patients with sepsis and septic shock are significantly associated with high mortality [27]. As cell-mediated immunity (CD4 T-helper-response and interferon (INF)-gamma) play an essential role in *M. tuberculosis* infection [28, 29], any impairment in T cell proliferation due to reduced leptin concentrations may contribute to the development of tuberculosis. In our patient group, both before and after treatment, leptin levels were suppressed, supporting the concept that leptin induction represents a protective component of the immune response in pulmonary tuberculosis. Conflicting data have been reported for leptin levels during infection. Eleveted leptin levels were detected in the studies published by Çakir et al. [23] and Yüksel et al. [30]. On the other hand, Van Crevel et al. [24] have shown that both plasma leptin and ex vivo IFN-gamma production were low and increased upon successful antituberculous treatment. Schwenk et al. [31] did not find any correlation between serum IL-6, TNF-*α*, and leptin levels.

In the present study, we could not find any correlation between TNF-*α*, leptin concentration, and chest X-ray findings. Tsao et al. [32] studied bronchoalveolar lavage (BAL) fluid and serum TNF-*α*, IL1*β*, and TNF-*α* receptor levels in patients with cavitated and noncavitated tuberculosis. The BAL fluid TNF-*α*, IL1*β*, and TNF-*α* receptor levels were higher in patienst with cavitated tuberculosis, but there was no such difference in serum levels. We also could not find any difference in the serum leptin concentration and TNF-*α* levels of cavitated and noncavitated tuberculosois patients. On the control chest X-rays obtained after anti-TB therapy, sequele lesions lie chronic fibrotic changes were detected in 19 patients. There was no difference in the ESR, BMI, and TNF-*α* levels of patients with and without sequele, but the leptin concentration was significantly low in patients with sequele. We presume that the sequelae lesions are the result of reduced inflammatory response due to low leptin concentration. But we do not know the duration of low leptin concentration in patients with sequelae. We also do not know whether low leptin concentration has any contribution in patients with relapse. We believe that further studies are needed to answer these questions.

In conclusion, leptin release is suppressed in tuberculosis and probably low leptin concentrations may contribute to the increased infection susceptibility and recovery with sequele lesions in patients with tuberculosis.

## Figures and Tables

**Table 1:** Demographic characteristics of patients with tuberculosis.

Number	25
Sex	19 men, 6 women
Age, (mean ± SD) years	36 ± 14.66
Chest X-ray
Minimal (%)	8(%32)
Moderate-advanced (%)	10(%40)
Far-advanced (%)	7(%28)
Symptoms and findings
Weight loss (%)	17(%68)
Appetite (%)	12(%48)
Body mass index mean ± SD (kg/m^2^)	19.36 ± 2.55
Body temperature, mean ± SD°C	37.16 ± 0.89

**Table 2:** Age, ESR, BMI, leptin, and TNF-*α* in controls and patients before and after tuberculosis therapy. Data presented as mean ± SD.

Parameters	Patients		Controls
	Before therapy	After therapy	

Age	36 ± 14.66	—	34.22 ± 12.9
BMI (kg/m^2^)	19.36 ± 2.55*	22.87 ± 3.13	24.23 ± 3.56
ESR (mm/h)	62.04 ± 26.96	12.64 ± 8.8^†^	(Male < 25, female < 30)
Leptin (ng/mL)	1.66 ± 1.68^¶^	3.26 ± 3.81^‡^	15.74 ± 9.12
TNF-*α* (pg/mL)	23.19 ± 12.78^§^	15.95 ± 6.58	14.22 ± 7.17

Comparison of case and control groups before therapy: *P < .05, ^¶^ *P* = .001, ^§^ *P* = .01.

Comparison of case and control groups after therapy: ^‡^ *P* = .001.

Comparison of ESR before and after therapy: ^†^ *P* = .01.

**Table 3:** TNF-*α* and leptin levels according to chest X-ray scores. Data presented as mean ± SD.

	TNF-*α*			Leptin		
	Before therapy	After therapy	*P*	Before therapy	After therapy	*P*

Minimal (*n* = 8)	22.07 ± 8.04	16.18 ± 7.64	NS	2.39 ± 2.57	5.67 ± 5.60	< .05
Moderate-advanced (*n* = 10)	22.44 ± 12.19	17.46 ± 6.01	NS	1.37 ± 1.06	3.32 ± 2.0	< .05
Far-advanced (*n* = 7)	25.54 ± 18.61	13.54 ± 6.36	NS	1.24 ± 0.89	1.15 ± 0.88	NS
*P* *	NS	NS	—	NS	NS	—

*Comparison between three groups of TNF and leptin levels.

NS = not significant *P* > .05.

**Table 4:** Serum leptin and TNF-*α* levels in patients with sequelae lesions and cavitary tuberculosis. Data presented as mean ± SD.

	Sequelae lesion	No sequelae lesion	Cavity	No cavity
	*n* = 6	*n* = 19	*n* = 7	*n* = 18

Age	36.22 ± 16.43	35.42 ± 9.76	35.71 ± 18.40	36.11 ± 13.56
BMI	19.0 ± 2.74	20.3 ± 1.86	18.20 ± 3.71	19.82 ± 1.89
BMI*	22.42 ± 3.21	24.01 ± 2.79	22.0 ± 4.89	23.21 ± 2.22
Leptin	1.29 ± 0.85	2.61 ± 2.79	1.52 ± 1.13	1.71 ± 1.87
Leptin*	2.13 ± 1.96	6.9 ± 5.72^¶^	3.06 ± 3.54	3.62 ± 4.19
TNF-*α*	22.1 ± 13.07	25.98 ± 12.54	21.36 ± 11.15	23.9 ± 13.60
TNF-*α* *	16.49 ± 6.65	14.57 ± 6.70	14.44 ± 6.56	16.54 ± 6.69

^¶^ *P* < .05.

*Levels of parameters after antituberculosis therapy.
